# Kinetics and minimal inhibitory concentrations of ceftiofur in tear film following extended-release parenteral administration (Excede^®^) in dogs

**DOI:** 10.3389/fvets.2022.975113

**Published:** 2022-09-20

**Authors:** Anna Catherine Bowden, Rachel A. Allbaugh, Joe S. Smith, Jonathan P. Mochel, Lionel Sebbag

**Affiliations:** ^1^Department of Veterinary Clinical Sciences, College of Veterinary Medicine, Iowa State University, Ames, IA, United States; ^2^Department of Biomedical Sciences, SMART Pharmacology, College of Veterinary Medicine, Iowa State University, Ames, IA, United States; ^3^Koret School of Veterinary Medicine, Hebrew University of Jerusalem, Rehovot, Israel

**Keywords:** bacterial keratitis, antibiotic, tear film, canine, PK-PD, blood tear barrier, ceftiofur

## Abstract

**Purpose:**

Describe the pharmacokinetics of extended-release parenteral ceftiofur (Excede^®^) in canine tear film and compare these concentrations to minimal inhibitory concentrations (MICs) of ceftiofur against common ocular pathogens in dogs.

**Method:**

Six dogs of various breeds were enrolled. Disruption of blood-tear barrier was achieved with histamine-induced conjunctivitis to ensure clinical relevance of the results. Each dog received a single subcutaneous injection of 5 mg/kg Excede^®^, followed by tear collection with Schirmer strips at times 0, 0.25, 0.5, 1, 2, 4, 8, 12, 24, 36, 48, 72, 96, 120, 144, 168, 192, 216 and 240 h. Drug quantification was performed with liquid chromatography-mass spectrometry. MICs were determined for *Staphylococcus pseudintermedius, Streptococcus canis* and *Pseudomonas aeruginosa* by assessing bacterial growth (*n* = 10 per bacterial species) in the presence of ceftiofur at increasing concentrations.

**Results:**

Blood-tear barrier breakdown provided tear film concentrations of ceftiofur 3.2–28.9-fold higher than in the contralateral healthy eye (*n* = 1 dog, pilot experiment). In all six dogs, ceftiofur concentrations in tears varied from 2.3 to 637.5 ng/mL and were detectable up to 10 days (240 h) after subcutaneous injection. However, tear levels always remained below MICs for common ocular isolates (≥640 ng/mL).

**Conclusions:**

Ceftiofur reached the tear compartment (for up to 10 days) after a single parenteral injection, however tear concentrations were extremely variable and too low to be effective against common bacterial pathogens in dogs. Further studies with different ceftiofur dosage or other long-acting injectable antibiotics are warranted.

## Introduction

Systemically administered medications can often serve as adjunctive therapy in the management of ocular surface diseases. Potential benefits of systemic therapy include ease of administration, improved owner/patient compliance, and possibly sustained drug levels when compared to topical instillation (due to rapid drug loss *via* nasolacrimal drainage, tear volume turnover, and mechanical forces from eyelid blinking) (1–3). Although promising, tear film levels are only described for select (oral or parenteral) medications in veterinary medicine such as doxycycline (4–6), minocycline, (7) pradofloxacin (6), famciclovir (8), voriconazole (9) and prednisone (10). Unfortunately, tear film pharmacology cannot be extrapolated from one drug to another given unique physicochemical properties (*e.g*., molecular weight, lipophilicity, protein binding) that influence drug distribution from the blood to tear film or other peripheral compartments; for instance, pradofloxacin but not doxycycline was quantifiable in tear film of cats receiving the same oral dose (5 mg/kg) (6). Further, tear film pharmacology cannot be extrapolated from one animal species to another, partly due to species differences in ocular surface anatomy and physiology (11); for instance, doxycycline is quantifiable in tear film of dogs (4, 5) but not cats (6).

Ceftiofur, a third-generation cephalosporin, is a β-lactam antibiotic that is labeled for use in several veterinary species (12). Ceftiofur crystalline-free acid (Excede^®^), an extended-release injectable formulation of ceftiofur, was recently investigated for “off label” use in dogs for treatment of common bacterial infections. In that study, dogs received a single subcutaneous injection of ceftiofur crystalline-free acid (5 mg/kg) that achieved quantifiable plasma concentrations for up to 10 days as well as ceftiofur levels above minimal inhibitory concentrations (MICs) of common respiratory/integumentary/urinary pathogens for several days (12). In another canine study, ceftiofur was shown to be effective against common bacterial pathogens isolated from dogs with infectious keratitis (13); *in vitro* susceptibility of 352 ocular isolates to ceftiofur was 79% overall, ranging from 20% (*Pseudomonas sp*.) to 88% (*Staphylococcus sp*.) and 99% (*Streptococcus sp*.). As such, ceftiofur may be useful for managing patients with bacterial keratitis, although tear film pharmacokinetics following parenteral administration have not been studied to date in any species.

The first objective of this study was to characterize tear film pharmacokinetics of ceftiofur following subcutaneous administration of a sustained-release formulation (ceftiofur crystalline-free acid) in dogs. The second objective was to determine the MICs of ceftiofur against common ocular pathogens in dogs, allowing for a preliminary pharmacokinetic-pharmacodynamic (PK-PD) analysis of subcutaneous ceftiofur as a potential therapeutic option for canine patients with bacterial keratitis. Notably, results were made as clinically relevant as possible by (i) assessing tear film concentrations in the presence of compromised blood-tear barrier, (10) and (ii) assessing MICs in the presence of albumin to account for protein-binding in tears (14).

## Materials and methods

### Tear film pharmacokinetics

Six dogs were enrolled, all confirmed to be healthy based on physical and ophthalmic examinations (slit lamp biomicroscopy (SL-17; Kowa Company, Ltd., Tokyo, Japan) of the adnexa and anterior segment, as well as indirect ophthalmoscopy (Keeler Vantage; Keeler Instruments, Inc., Broomall, PA, USA). Five dogs were spayed females while one dog was castrated male, the canine breeds were varied (3 mixed breed dogs, 1 Labrador Retriever, 1 German Shepherd, and 1 Shih Tzu), with a mean ± standard deviation age and body weight of 5.3 ± 3.1 (2–10) years and 19.4 ± 11.7 (6–31.8) kg. The sample size (*n* = 6) was based on the minimum number of animals described in previous studies assessing tear film pharmacokinetics following parenteral drug administration (4, 10), a sample deemed sufficient to estimate the main pharmacokinetic outcomes. The study was approved by the Institutional Animal Care and Use Committee of Iowa State University (IACUC-20-077).

#### Pilot experiment

Tear film concentrations of ceftiofur were evaluated in one dog in the presence or absence of conjunctival inflammation, as previously described (10). Briefly, 1% histamine ophthalmic solution was formulated by mixing histamine powder (histamine dihydrochloride, FCC grade, Acros^®^ Organics, Geel, Belgium) with 1.4% polyvinyl alcohol lubricating eye drops (Artificial tears solution; Rugby, Rockville Center, NY) in a sterile manner under a laminar flow hood, adjusting the pH to 6.5 to reduce ocular irritation. Twenty minutes before ceftiofur administration (5 mg/kg subcutaneously once) and before each tear collection, a single drop of histamine solution was applied to the left eye (inducing moderate conjunctivitis in a non-invasive and self-resolving manner) (15), while the other eye received artificial tears (Control). This concentration of histamine was chosen to induce reliable, moderate conjunctivitis with minimal to no discomfort to the study subject. Tear fluid was sampled simultaneously in both eyes at 0.5, 1, 2, 4, 8, 12, and 24h following ceftiofur administration. The bent tip of a Schirmer tear strip (Eye Care Product Manufacturing, LLC, Tucson, AZ) was placed in the ventrolateral conjunctival fornix of each eye until the 20-mm mark of wetness was reached. The distal portion of each strip (25–35 mm marks, not wetted with tears) was spiked with 5 μL internal standard (d3-ceftiofur; Toronto Research Chemicals, North York, Canada) prepared as 0.5 ng/μL solution in 1:1 acetonitrile:water (11), and samples were stored at −20°C until further analysis.

#### Full experiment

The full experiment was conducted >1 month after the preliminary study to allow for complete washout of ceftiofur in the ‘pilot' dog. Owing to results of the pilot experiment (see Results section), conjunctivitis was incorporated in the study design such as results can be made more clinically relevant. In each dog, one eye was randomly selected (using the Excel software 2016) to receive topical histamine and undergo sequential tear collections following ceftiofur administration; the other eye (uninflamed) was not sampled due to financial limitations in the number of biological samples that could be analyzed for drug content. Following a single subcutaneous injection of 5 mg/kg ceftiofur crystalline-free acid (Excede^®^ for swine, Zoetis, Florham Park, NJ), tear fluid was sampled (as described above) at 0.25, 0.5, 1, 2, 4, 8, 12, 24, 36, 48, 72, 96, 120, 144, 168, 192, 216, and 240h (10 days). At each session, the eye received 1 drop of 1% histamine solution 20 min prior to tear collection, allowing conjunctivitis to develop (confirmed with gross examination of the eye) and ocular surface homeostasis to be restored; (16) tear fluid was then collected with Schirmer strips (until 20-mm mark was reached, recording the collection duration with a stopwatch), 5 μL of d3-ceftiofur internal standard was spiked on the distal (dry) portion of the strip, and samples were stored in 1.5 mL Eppendorf tubes at −20°C until analysis.

#### Ceftiofur quantification in tears

Given the unstable nature of ceftiofur (i.e., rapidly degraded to desfuroylceftiofur and furoic acid) (17), samples were analyzed following cleavage of all ceftiofur related residues (parent, metabolites, and protein bound residues) to desfuroylceftiofur (DFC) and subsequent derivatization to desfuroylceftiofur-acetamide (DCA). In practice, Schirmer strips were thawed at room temperature, soaked with 750 μL of 0.5% dithioerythritol in borate buffer (0.2 Molar, pH 9), kept in +4 refrigerator for 45 min then placed in a water bath at 50°C for 15 min. Then, 150 μL of 14% iodoacetamide solution was added and the samples were placed in the dark to complete the derivatization process to DCA. Of note, the internal standard (d3-ceftiofur) was transformed into d3-DCA upon cleavage and derivatization.

Blank canine tears obtained using ophthalmic sponges (18) (leftovers from a previous study in Beagle dogs) (10) were used for the calibration samples, defrosted from −80°C freezer prior to use. Eight standard curve solutions (1, 2, 5, 10, 20, 50, 100 and 200 ng/mL) and three quality control samples (15, 80, 150 ng/ml) were prepared by spiking blank canine tears with stock solutions of ceftiofur (ceftiofur analytical standard, Sigma Aldrich, Milwaukee, WI), transferring 18 μL of each solution onto separate Schirmer strips (18 μL ~ 20-mm mark wetness) (19), spiking 5 μL of d3-ceftiofur internal standard on the distal (dry) portion of the strips, then processing the calibration samples in a similar fashion than biological samples.

DCA residues were measured by LC-MS/MS analysis following solid phase extraction (SPE) of derivatized tears samples on Oasis HLB cartridges (Waters, Milford, MA). The SPE clean-up was conducted on 1 cc, 30 mg HLB cartridges. A rinse of 0.5 mL of 5% methanol in water was performed prior to elution with two 0.5 mL portions of 5% acetic acid in acetonitrile. After dry-down at 40°C in a Turbovap, the tube contents were reconstituted with 50 μL of 25% acetonitrile followed by 75 μL of water. The samples were transferred to autosampler vials fitted with 300 μL glass inserts and then centrifuged at 2,000 x g prior to analysis.

The UHPLC analysis was conducted with an UltiMate 3000 Pump, Column Compartment and Autosampler (Thermo Scientific, San Jose, CA, USA) coupled to an Orbitrap mass spectrometer (Q Exactive Focus, Thermo Scientific, San Jose, CA, USA). The chromatographic column was an ACE UltraCore 2.5 Super C18 column (100 x 2.1 mm id, MAC-MOD Analytical, Chads Ford, PA). The column temperature was 45°C and the autosampler was maintained at 10°C. Mobile phases A and B were 0.1% formic acid in LC-MS grade water and methanol, respectively. The solvent gradient was from 5% methanol to 95% methanol in 6 min at 0.325 mL/min with a two-minute re-equilibration at 0.45 mL/min. Parallel reaction monitoring in the positive electrospray ion mode was used for analyte detection with a spray voltage of 4.0 kV and a temperature of 350°C. The precursor ions were determined by the instrument software from the molecular formulas. These were DCA C16H18N6O6S3 or m/z of 487.0523 and d3-DCA C16H15D3N6O6S3 or m/z 490.0711. A collision energy of 25 electron volts (eV) was used for fragmentation of all the analytes within the collision cell. Five fragment ions were used for quantitation of DCA. These fragment ions were at 167.027, 210.020, 241.039, 285.010 and 324.057 m/z, while ions at 244.057, 288.029, and 327.076 m/z were characteristic of d3-DCA fragmentation.

Quantitative results were obtained by batch processing the raw MS data of each sample in sequences of tear blanks, calibration spikes, and canine tear samples through a processing method that identified and integrated each peak in each sample and calculated the internal standard based calibration curve using a weighted (1/X) linear fit. Tear concentrations of DCA in unknown samples were calculated by the Xcalibur software (version 2.3.1, Thermo Fisher Scientific) based on the calibration curve. Results were then viewed in the Quan Browser portion of the Xcalibur software. The limit of quantitation (LOQ) of the analysis was 1.0 ng/mL for DCA (the lowest calibration spike) with a limit of detection (LOD) of 0.2 ng/mL. The accuracy of the method and the analytical runs was accepted with a bias of the nominal concentrations within ±8%, the precision was sufficient with values for the coefficient of variation (CV) within ±15% and the linearity was adequate with a correlation coefficient (r^2^) exceeding 0.992.

### Minimal inhibitory concentrations

This *in vitro* experiment was performed in concert with the above experiment and focused on the 3 most common bacterial species identified in dogs with infectious keratitis in the local area, that is, *Staphylococcus pseudintermedius, Streptococcus canis*, and *Pseudomonas aeruginosa* (13). Ten bacterial isolates were selected for each bacterial species. Bacterial isolates (*n* = 30) cultured from canine patients were revived from the −80°C freezer by thawing at room temperature and grown on tryptic soy with 5% sheep blood agar plates. Bacterial broths containing 4 × 10^7^ colony forming units per milliliter (CFU/mL) were prepared using Mueller-Hinton broth (MHB; catalog #T3462, Thermo Scientific Inc.) for *Staphylococcus pseudintermedius* and *Pseudomonas aeruginosa*, or Mueller-Hinton broth with lysed horse blood (catalog # CP114-10, Thermo Scientific Inc.) for *Streptococcus canis*, as previously described (14). Albumin 4% solution was prepared by mixing canine albumin powder (Animal Blood Resources International, Stockbridge, MI) with deionized water. Similarly, ceftiofur solutions were prepared by mixing ceftiofur (Vetranal^®^, Sigma Aldrich, Milwaukee, WI) with deionized water to obtain the following concentrations: 40 ng/mL, 80 ng/mL, 160 ng/mL, 320 ng/mL, 640 ng/mL, 1,280, 2,560, and 5,120 ng/mL.

Using one blank plate (Corning 96-well Clear Polystyrene Microplates, Corning Inc.) for each bacterial species, 15 μL of albumin solution was pipetted into 80 wells (10 columns x 8 rows), 15 μL of a given bacterial isolate was pipetted in the 8 wells of a given column (*e.g*., column 1 for isolate #1, column 2 for isolate #2, …., column 10 for isolate #10), and 30 μL of a given ceftiofur concentration was pipetted in the 10 wells of a given row (*eg*, row 1 for 40 ng/mL, row 2 for 80 ng/mL, …, row 8 for 5,120 ng/mL). Accounting for the dilutions resulting from mixing the 3 different solutions in the wells, bacterial load was set to the standard for susceptibility testing (10^7^ CFU/mL), albumin level was set to 1% (average albumin levels in canine tear film), (14, 15) and ceftiofur concentrations ranged from 20 ng/mL to 2,560 ng/mL.

Following CLSI guidelines, plates were incubated at 37°C ± 2°C for either 16–20 h (*Pseudomonas sp*.), 20–24 h (*Streptococcus sp*.), or 24 h (*Staphylococcus sp*.), followed by data recording using a digital MIC viewing system (Sensititre Vizion^TM^, Thermo Scientific Inc.). The presence or absence of bacterial growth was recorded in each well.

### Data analysis

For each bacterial species, MIC_50_ and MIC_90_ were read as the lowest concentration of ceftiofur that completely inhibited bacterial growth in 50% and 90% of the bacterial isolates, respectively (20). The time ceftiofur tear concentrations were above MICs (T > MIC), a key pharmacokinetic-pharmacodynamic (PK-PD) parameter to predict clinical efficacy of a β-lactam antibiotic such as ceftiofur, were recorded. A Spearman's correlation test was used to assess for potential association between tear film ceftiofur concentration and tear flow rate (determined by dividing tear volume collected by the duration of tear collection), (4) and between tear film ceftiofur concentrations and body weight. Statistical analyses were performed with SigmaPlot 14.5 (Systat software, Point Richmond, CA), and *P* < 0.05 were considered as statistically significant.

## Results

Table 1 summarizes the results of the pilot experiment. Overall, tear film concentrations of ceftiofur were higher in the eye with histamine-induced conjunctivitis (compromised blood-tear barrier) compared to the contralateral healthy eye at all time points investigated, with the fold change between eyes ranging from 3.2 to 28.9.

**Table 1:** Tear film concentrations of ceftiofur in one dog (pilot experiment) with histamine-induced conjunctivitis in one eye and a healthy contralateral eye.

	**Tear film ceftiofur concentrations (ng/mL)**		
**Time (hr)**	**Control eye**	**Conjunctivitis eye**	**Fold**
			**difference**
0.5	2.3	14.1	6.1
1.0	1.8	21.0	11.7
2.0	1.0	5.7	5.7
4.0	1.0	7.8	7.8
8.0	5.2	47.7	9.2
12.0	3.4	97.6	28.9
24.0	18.6	58.9	3.2

**Figure 1:**
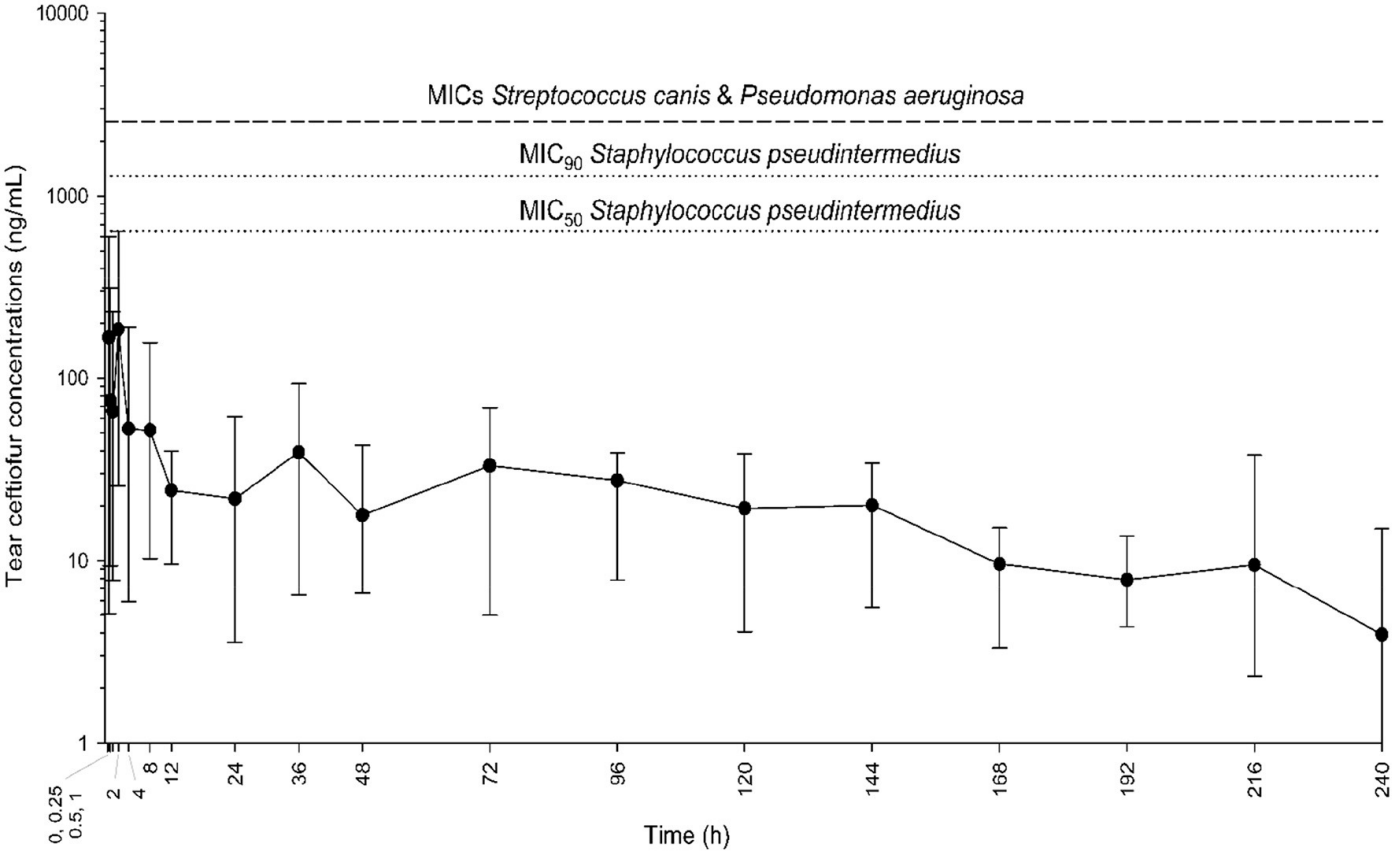
Mean and range (minimum-maximum) tear film concentrations of ceftiofur over time (0.25–240 h) in six dogs receiving a single subcutaneous injection of 5 mg/kg ceftiofur crystalline-free acid at baseline. Eyes were inflamed (histamine-induced conjunctivitis) to better mimic clinical scenarios (i.e., breakdown of blood-tear barrier in diseased eyes). Horizontal dotted lines represent the minimal inhibitory concentrations (MICs) for common bacteria isolated from dogs with infectious keratitis (*Staphylococcus pseudintermedius, Streptococcus canis, Pseudomonas aeruginosa*).

In all six dogs, ceftiofur concentrations in tears varied from 2.3 to 637.5 ng/mL and were detectable up to 10 days after the single subcutaneous injection of ceftiofur crystalline-free acid (Figure 1). Maximal ceftiofur concentrations were detected at t = 2 h post subcutaneous dosing [mean (range) tear film ceftiofur levels = 184.6 (25.7–637.5) ng/mL], followed by a slow decrease in tear film concentrations over the 10-d study period. Tear film ceftiofur concentrations were not associated with body weight (r = 0.11, *P* = 0.239) nor flow rate during tear collections (r = 0.17, *P* = 0.157).

For *Staphylococcus pseudintermedius* isolates (*n* = 10), MICs of ceftiofur ranged from 640 ng/mL to 2,560 ng/mL, with a MIC_50_ of 640 ng/mL and MIC_90_ of 1,280 ng/mL. However, for all isolates of *Streptococcus canis* and *Pseudomonas aeruginosa* (*n* = 20), none of the ceftiofur concentrations tested were sufficient to inhibit bacterial growth; therefore, the MICs for these two bacterial species were recorded as > 2,560 ng/mL. Time above MICs was 0 h, that is, tear film levels remained below MICs (of all isolates) at all time points investigated.

## Discussion

Ceftiofur was quantifiable in the canine tear film for up to 10 days following a single subcutaneous injection of ceftiofur crystalline-free acid in dogs. Similar to previous reports with doxycycline, (4) tear film concentrations were not affected by the dog's body weight nor the flow rate during tear collection with Schirmer strips. Further, tear film concentrations fluctuated over time with tear levels initially decreasing from 0.25 to 1 h post- dosing, increasing to reach maximal levels at 2 h post- dosing, then gradually decreasing until the last time point measured (240 h). This ‘non-linear' kinetic profile was also demonstrated for other systemic antibiotic in cats (i.e., pradofloxacin) (6) and cattle (i.e., chloramphenicol, erythromycin, gentamicin and oxytetracycline), (21) and may be due to the underlying mechanism therapeutic drugs reach the tear film from the blood compartment (i.e., passive/transient diffusion through conjunctiva ± drug reservoir in the lacrimal glands). In fact, the kinetic profile of ceftiofur in blood differs from the one in the tear film compartment following parenteral drug administration. Although plasma levels of ceftiofur were not evaluated in the present study, comparison with another group of dogs receiving the same dose of ceftiofur (5 mg/kg) (12) highlights two interesting findings: (1) Tear film concentrations reached ~ 9.3% of plasma levels (based on C_max_ values), a value that is consistent with the tear-to-plasma ratio (12%) for another antibiotic recently tested in dogs (doxycycline); (4) and (2) time to reach maximal concentration was faster in tears than in plasma (2 h vs. 23.2 h, respectively). The latter finding was also described in dogs that were administered oral prednisone at a given dosage (0.5 mg/kg), (10, 22) yet the potential explanation remains puzzling as one would expect T_max_ to be consistently longer in tears when compared to the blood compartment, regardless of the drug or the dosage. This finding may be partly explained by the high variability in drug quantification inherent to tear fluid (i.e., disturbance of ocular surface homeostasis, depletion of tear fluid during tear sample collection), making calculations of T_max_ less reliable than in plasma or other bodily fluids.

Tear film ceftiofur levels were below MICs of common ocular pathogens in dogs, suggesting that subcutaneous ceftiofur administration at 5 mg/kg is likely ineffective in the management of canine patients with bacterial keratitis. This finding differs from ceftiofur efficacy in cattle, where parenteral ceftiofur is known to be effective in clinical patients with infectious bovine keratitis (23). Differences in bacterial susceptibility (i.e., greater ceftiofur efficacy against *Moraxella bovis* compared to canine isolates) and/or tear film pharmacokinetic profiles (tested in dogs but not cattle) may explain differences in ceftiofur efficacy between species. This finding is also surprising as ceftiofur concentrations reported by Hooper et al. were above MICs for common canine pathogens, including *Staphylococcus pseudintermedius* (MICs as low as ≤ 0.25 μg/mL) (12, 24). Notably, MICs calculated in the present study somewhat differed from previous canine reports, likely owing to the pathogen selection (cornea *vs*. other body locations) and the incorporation of serum albumin in the current study's test media. For *Staphylococcus pseudintermedius*, MICs varied from 0.64 to 2.56 μg/mL compared to ≤ 0.25 to ≥8.0 μg/mL in previous experiments (12, 24). MICs for *Pseudomonas aeruginosa* were > 2.56 μg/mL compared to 0.5 to ≥8.0 μg/mL in previous studies (12, 25). MICs for *Streptococcus canis* (> 2.56 μg/mL in present work) were not specifically evaluated to date (26).

Oral or parenteral administration of therapeutic drugs represent a promising tool for adjunct therapy of ocular surface diseases owing to several key advantages. While the ocular bioavailability of topical administration is poor given efficient washout by tears (1–3), parenteral administration could be considered as a form of sustained release at the ocular surface through lacrimal gland diffusion and conjunctival leakage (10). Further, systemic drug administration could help improve patient (27) and owner compliance, especially in cases of bacterial keratitis where multiple medications have to be applied as often as every 1–2 h (28). Ceftiofur represented an ideal candidate for adjunct therapy of bacterial keratitis given the sustained-release profile of ceftiofur crystalline-free acid in dogs (12). The sustained-release formulation (Excede^®^) is recommended at a dose of 5 mg/kg in swine and 6.6 mg/kg in horses/cattle (Zoetis prescribing information); here we selected 5 mg/kg based on the only available “off-label” data in the canine species, showing that this dose was well tolerated in dogs and provided systemic drug levels for up to 10 days after a single subcutaneous administration. It is possible that a larger dose (> 5 mg/kg) or repeated administration of ceftiofur crystalline-free acid would provide superior PK-PD profiles in the canine tear film; however, the high protein binding of ceftiofur in plasma (94%) (29) may limit the amount of drug diffusion from blood to tear fluid regardless of the plasma concentrations. Investigation of other systemic antibiotics are warranted in dogs, especially ones with different physicochemical properties (e.g., protein binding, lipophilicity, molecular weight) that could potentially achieve sufficient drug levels in the tear film to inhibit the growth of common ocular pathogens in dogs. In doing so, it seems important to account for important physiological changes that occur in clinical patients, namely:

- ***Blood-tear barrier breakdown:*** Dogs with ulcerative keratitis (or other inflammatory conditions) often develop concurrent conjunctivitis as bystander inflammation, a condition that promotes leakage of compounds from the blood-to-tear compartment (15, 30). This physiological process was shown in several studies for large proteins such as albumin (11, 14, 15, 31) but could also be true for smaller compounds such as xenobiotics. In the present study, tear film concentrations of ceftiofur were higher in the canine eye with conjunctivitis compared to the contralateral healthy eye at all time points investigated (0.5–24 h), with a difference in tear concentrations up to ~29-fold. In other words, pharmacokinetic studies conducted in healthy eyes likely underestimate the true drug concentrations present in clinical patients, leading to erroneous conclusions about drug efficacy or lack thereof. One should consider a pilot experiment (as conducted herein) as the effect of conjunctivitis may vary from one drug to another–for instance, ceftiofur levels were greatly affected by conjunctivitis in the present study, but this was not the case for prednisone/prednisolone in another experiment (10).
- ***Antibiotic-protein binding in tear film:*** Diseased eyes have elevated levels of albumin in the tear film, leading to protein-antibiotic binding that could modulate drug efficacy on the ocular surface. In one study, albumin levels > 0.05% increased MICs of canine ocular pathogens in a dose-dependent, bacteria-specific, and antibiotic-specific manner (14). Here, 1% canine albumin was added to bacterial wells to optimize *in vitro* susceptibility testing with *in vivo* conditions.

There are several limitations to our study. First, the canine population was not homogenous. Dogs of various breeds and sizes were enrolled in an attempt to better represent clinical patients managed by veterinarians (i.e., bacterial keratitis is more common in Shih Tzu *vs*. Beagle dogs); however, this choice may have introduced variability in the pharmacokinetic data owing to differences in ocular surface physiology and tear film dynamics (e.g., tear volume) (32). Second, MICs were calculated using only *n* = 10 isolates for each bacterial species; this number was sufficient to determine MIC_50_ and MIC_90_ but insufficient to generalize findings to the general population. If the selected bacterial isolates were somewhat ‘resistant' strains compared to the general susceptibility profile, actual MICs on larger sample size (i.e., 100+ isolates per bacterial species) might have been lower and the resulting PK-PD outcome (time > MIC) might have been more favorable. Third, histamine-induced conjunctivitis–used to better mimic clinical scenarios *via* a reliable, non-invasive, and self-resolving manner (15)–might have affected tear film concentrations by disturbing ocular surface homeostasis (*e.g*., reflex tearing, accelerated nasolacrimal drainage). Care was taken to minimize the potential impact of histamine by delaying tear collection (20 min from each histamine administration), thereby allowing ocular surface homeostasis to be fully restored; (16) nonetheless, results of the present experiment should be verified in canine patients with bacterial keratitis and associated *naturally occurring* conjunctivitis. Last, the study focused on canine eyes with avascular corneas. As such, the speculation that Excede^®^ is inefficient is dogs with bacterial keratitis may not be true in dogs with vascularized corneal ulcers or dogs undergoing surgical stabilization with conjunctival grafting. In fact, the latter may benefit from systemic antibiotherapy as recently shown by Osinchuk and colleagues (33).

In conclusion, ceftiofur administered subcutaneously at 5 mg/kg reached the tear fluid of dogs (for up to 10 days) after a single injection of a sustained-release formulation (Excede^®^). However, tear concentrations were too low to be effective against common bacterial pathogens in dogs. Further studies with different ceftiofur dosing regimen, or other long-acting injectable antibiotics are warranted before systemic therapy could complement topical therapy in companion animals with bacterial keratitis. Given the costs involved with pharmacokinetic studies and the ethical considerations of *in vivo* experiments in animals, investigators could first screen for the most suitable drugs (*in vitro* MIC testing using ocular isolates and a range of estimated drug concentrations) then move forward with parenteral administration and drug quantification in tear film of companion animals.

## Data availability statement

The raw data supporting the conclusions of this article will be made available by the authors, without undue reservation.

## Ethics statement

The animal study was reviewed and approved by the Institutional Animal Care and Use Committee of Iowa State University. Written informed consent was obtained from the owners for the participation of their animals in this study.

## Author contributions

LS conceptualized and designed the study in consultation with JM. AB, RA, JS, and LS performed the experiments. LS and JM analyzed the data. All authors wrote the manuscript, contributed to the article, and approved the submitted version.

## Conflict of interest

The authors declare that the research was conducted in the absence of any commercial or financial relationships that could be construed as a potential conflict of interest.

## Publisher's note

All claims expressed in this article are solely those of the authors and do not necessarily represent those of their affiliated organizations, or those of the publisher, the editors and the reviewers. Any product that may be evaluated in this article, or claim that may be made by its manufacturer, is not guaranteed or endorsed by the publisher.
